# Discovery of the biostimulant effect of asparagine and glutamine on plant growth in *Arabidopsis thaliana*

**DOI:** 10.3389/fpls.2023.1281495

**Published:** 2024-01-22

**Authors:** Manon Lardos, Anne Marmagne, Nolwenn Bonadé Bottino, Quentin Caris, Bernard Béal, Fabien Chardon, Céline Masclaux-Daubresse

**Affiliations:** ^1^ Université Paris-Saclay, INRAE, AgroParisTech, Institut Jean-Pierre Bourgin (IJPB), Versailles, France; ^2^ NOVAEM, Aigrefeuille d’Aunis, France

**Keywords:** protein hydrolysate, *in vitro* experiment, amino acid enantiomer, amino acid use efficiency, *Arabidopsis thaliana*

## Abstract

Protein hydrolysates have gained interest as plant biostimulants due to their positive effects on plant performances. They are mainly composed of amino acids, but there is no evidence of the role of individual of amino acids as biostimulants. In this study we carried out *in vitro* experiments to monitor the development of Arabidopsis seedlings on amino acid containing media in order to analyze the biostimulant properties of the twenty individual proteinogenic amino acids. We demonstrated that proteinogenic amino acids are not good nitrogen sources as compared to nitrate for plant growth. Biostimulant analyses were based on leaf area measurements as a proxy of plant growth. We developed the Amino Acid Use Efficiency index to quantify the biostimulating effect of individual amino acids in the presence of nitrate. This index allowed us to classify amino acids into three groups, characterized by their inhibiting, neutral, and beneficial effects regarding leaf area. Glutamine and asparagine demonstrated the most significant effects in promoting leaf area in the presence of nitrate supply. The stimulating effect was confirmed by using the L and D enantiomeric forms. Both L-glutamine and L-asparagine stimulated leaf area at low concentrations, emphasizing their biostimulating properties. Our plant growth design and AAUE index pave the way for the identification of other bioactive molecules in protein hydrolysates and for the comparison of biostimulant performances.

## Introduction

In a context where the world’s population is estimated to be about 9.3 billion people by 2050 (Department of Economic and Social Affairs, 2022), agriculture must face the twin challenges of assuring food security and reducing pressure on the environment and natural resources (Searchinger et al., 2013). To date, the use of fertilizers essentially in the form of ammonium nitrate assures the stability of agricultural crop yield. However, in many countries, the overuse of those fertilizers is responsible for nitrogen leaching in groundwater (Sun et al., 2019) and for nitrous oxide emission (Billen et al., 2013). In addition, the synthesis of ammonium nitrate fertilizers through the Haber Bosch process is costly and fossil energy-consuming. To tackle these-substantial negative environmental consequences, it is crucial to identify and characterize sustainable inputs that can improve the plant nitrogen use efficiency.

Biostimulants constitute a suitable answer to this challenge. They are a novel category of agricultural inputs recently defined by the European Parliament. They stimulate nutrient use efficiency, tolerance to abiotic stress, quality trait, or availability of confined nutrients in the soil or rhizosphere independently of the product’s nutrient content (Regulation EU, 2019). Moreover, they participate in a circular economy allowing upcycling of by-products or waste from other industries (Baglieri et al., 2014; Colla et al., 2015). There are 7 classes of biostimulants; among them protein hydrolysates (PHs) are produced from strong acid or alkaline hydrolysis of plants, vegetable by-products, or animal sources (i.e. leather, viscera, feather, blood) (Colla et al., 2015; du Jardin, 2015; Xu and Geelen, 2018). Due to their mode of production PHs mostly contain amino acids (AAs) and small peptides. Their foliar or root application can promote the growth and yield of crops. For instance, PHs could enhance the yield of soybean and pepper by 32% and 22% respectively (Paradiković et al., 2011; Kocira, 2019). They could also enhance the root growth and fruit weight of tomatoes and the fresh biomass of lettuce (Cerdán et al., 2013; Rouphael et al., 2017; Malécange et al., 2022). The above positive effects might be associated with the stimulation of leaf sugar accumulation and nitrogen assimilation (Schiavon et al., 2008; Ertani et al., 2013). Nevertheless, despite plenty of examples of their positive effects and because of the diversity and inconsistency of source materials used for these products, the identity of the bioactive molecules of PHs remains to be determined.

In PHs from animal or vegetable sources, total amino acids can represent 27% to 68.5% of the total nitrogen content and individual amino acids vary from 2% to 18% of the total product depending on the sources of materials used to produce them (Calvo et al., 2014; Colla et al., 2014; Lucini et al., 2015; Ambrosini et al., 2021). It is well known that amino acids are essential molecules in plants as building blocks of proteins. They are also involved as precursors of tremendous specialized metabolites for plant adaptation to environmental stresses (Zhao, 2010; Maeda and Dudareva, 2012; Hildebrandt et al., 2015). Soil can contain free amino acids and plant roots possess transporters on plasma membranes for their absorption (Näsholm et al., 2009; Tegeder and Rentsch, 2010; Tegeder and Masclaux-Daubresse, 2018; Yang et al., 2020; Yao et al., 2020). Amino acid transporters described in literature belong to families as AAP (Amino acids permease), LHT (Lysine Histidine like transporter) and ProT (Proline transporter). Once they are absorbed by the plant, amino acids can be directly used for root growth or loaded to xylem to be transported to the aerial parts and contribute to general plant growth (Tegeder, 2014). Several publications report that amino acids alone are not efficient sources of nitrogen for plant growth. For example, Forsum et al. (2008) demonstrated that when *Arabidopsis thaliana* (Arabidopsis) seedlings were grown on amino acids, only six [Glutamine (Gln); Asparagine (Asn), Aspartate (Asp), Glycine (Gly), Alanine (Ala) and Arginine (Arg)] over the ten tested supported plant growth. However at equivalent supply of nitrogen, amino acids were weaker to promote growth compared to nitrate (Forsum et al., 2008). Nutritive effect of amino acids on plant growth is also dependent of their enantiomeric forms. Even if the L and D forms can be both absorbed by roots (Forsum et al., 2008), it seems that plants lack the capacity to metabolize the D-form in contrast to microbes (Bollard, 1966; Forsum, 2016). At the concentration of use described in literature, the positive effects of PHs cannot be explained by the nutritive effect of the amino acids they contain. Thus, we can hypothesize that the amino acids composing PHs display biostimulant effects.

In this study we examined the individual effects of the 20 proteinogenic amino acids on the growth of Arabidopsis seedlings. To discriminate biostimulant effect from nutritive effect, we first monitored leaf area when amino acids were provided as sole source of nitrogen. Next, we analyzed the positive, neutral or inhibitory effects of individual amino acids on plant growth, when provided in addition of a sufficient nitrate supply (KNO_3_). We then developed a new index AAUE (Amino acids use efficiency) as an indicator of the biostimulating effect of individual amino acids. The biostimulant activities of amino acids were then confirmed by decreasing the concentrations of amino acids supplied into the plant growth medium, and by using enantiomeric forms.

## Materials and methods

### Plant material and growth conditions

Seeds of the *Arabidopsis thaliana* Columbia wild type (*Col-0*) have been provided by the Versailles Resource center (INRA Versailles France, http://dbsgap.versailles.inra.fr/vnat/). *In vitro* culture was carried out using surface-sterilized seeds on horizontal agar plates. Using toothpick, seeds were sown on 0.8% agar medium with 1% sucrose, 2.5 mM KH_2_PO_4_, 2 mM MgSO_4_, 5 mM KCl, 2 mM CaCl_2_, 0.015 mM bromocresol purple (pH 5.8), 3.6 mM MES, 0.014 mM Fe-EDTA, supplemented with 1X microelements (Estelle and Somerville, 1987) and Morel and Wetmore vitamins. The concentrations of KNO_3_ and/or amino acids supplied in the media are indicated in the legends of the figures. All solutions had a pH of 5.8. After sowing, agar plates were incubated in a cold dark room at 4°C for 48 h for seed stratification and then transferred to a climate chamber (12 m^2^) under long day conditions (16/8 h photoperiod at 90 µmol photons m^-2^ s^-1^ with OSRAM LUMINUX COOL DAYLIGHT fluorescent lamps (L36W/865), 21°C day temperature and 18°C night temperature, relative humidity of 63%. Despite the global control of all the environmental parameters, there were still local uncontrolled variations due to the size of the chamber. To minimize related stochastic variation, the location of the plates in the growth chamber was changed regularly and all the experiments were repeated several times. Depending on experiments, the seed density was 16, 40, or 100 seeds per plate and is indicated in the legends of the figures. The culture duration was 12 days for high seed density and 14 days for low seed density.

### Leaf area imaging

All agar plates were imaged 12 or 14 days after sowing. Images were obtained using Gel doc Systems (Biorad^®^) with white trays. Then leaf area was determined using ImageJ software. (Version 1.53C).

### Determination of amino acid use efficiency

The contribution of an amino acid to plant growth was obtained by comparing the leaf area of the plants grown on plates containing amino acid and nitrate [AA + KNO_3_], nitrate alone [KNO_3_] or no nitrogen [0N]. To estimate contribution of individual amino acids to plant growth, we developed the AAUE index (Amino Acid Use Efficiency), that calculates the gain of growth in the presence of AA and nitrate compared to the gain in the presence of nitrate only, according to the equation below:


(1) $$AAUE=\frac{Area_{\left(AA+3N\right)}-{\bar{Area}}_{\left(Ctrl\;0N\right)}}{{\bar{Area}}_{\left(Ctrl\;3N\right)}-{\bar{Area}}_{\left(Ctrl\;0N\right)}}$$

with $Area_{\left(AA+3N\right)}$ is the leaf area of plants grown with amino acid and 3 mM nitrate in one plate, ${\bar{Area}}_{\left(Ctrl\;0N\right)}$ is the mean of leaf area of plants grown without nitrogen, ${\bar{Area}}_{\left(Ctrl\;3N\right)}$ is the mean of leaf area of plants grown with 3 mM nitrate only.

For plates corresponding to the control conditions (3 mM KNO_3_; Ctrl 3N) and (5 mM KNO_3_; Ctrl 5N), AAUE was calculated using the (**Equation 2**, **3**):


(2) $$AAUE=\frac{Area_{\left(3N\right)}-{\bar{Area}}_{\left(Ctrl\;0N\right)}}{{\bar{Area}}_{\left(Ctrl\;3N\right)}-{\bar{Area}}_{\left(Ctrl\;0N\right)}}$$

and


(3) $$AAUE=\frac{Area_{\left(5N\right)}-{\bar{Area}}_{\left(Ctrl\;0N\right)}}{{\bar{Area}}_{\left(Ctrl\;3N\right)}-{\bar{Area}}_{\left(Ctrl\;0N\right)}}$$

where Area_(3N)_ and Area_(5N)_ are the leaf area of plants grown with 3 mM KNO_3_; or with 5 mM KNO_3_ respectively.

In each experiment, 2 to 8 plates were prepared for each growth medium. On each plate, leaf area was estimated as the ratio of the total green area in the plate divided by the number of plants. We then calculated the mean of leaf areas for all the control conditions and computed the AAUE index by plate using the (**Equations 1**–**3**).

### Statistical analyses

**Figure 1:**
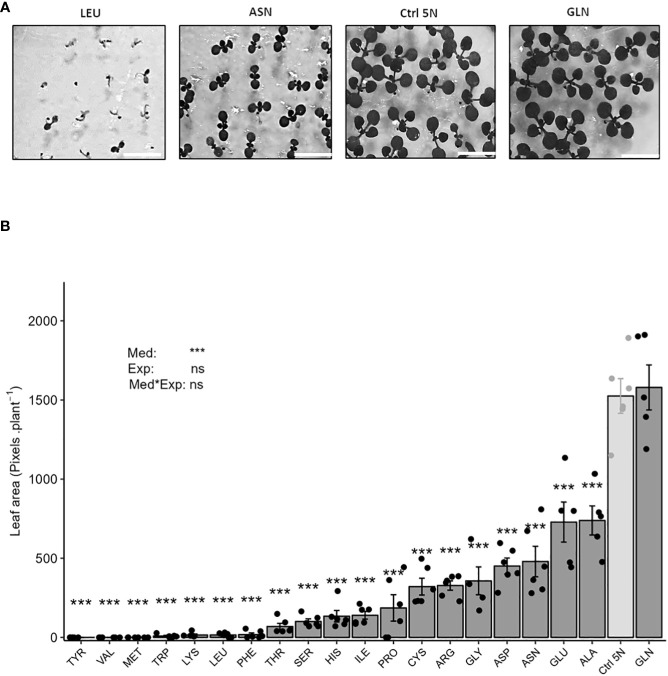
Glutamine and KNO_3_ are the best nitrogen sources for *in vitro* plant growth. Representative pictures of mediocre, weak and optimal plant growth phenotypes **(A)**, scale = 0.80 cm. Plant growth was determined measuring leaf area **(B)**. Seedlings (100 per plate) of the Col-0 accession were grown for 12 days on agar media containing 1% sucrose and 5 mM of one of the twenty proteinogenic amino acids. Media also contained 0.1 mM KNO_3_ to relieve dormancy and permit homogenous germination (Alboresi et al., 2005). The growth of the seedlings was determined by analyzing leaf area expressed in pixels per plant. The control consists of an agar medium containing 1% sucrose and 5 mM KNO_3_ (Ctrl 5N, light grey). Data represent mean values obtained in 3 independent experiments containing 0-2 repeats. Error bars indicate the standard error of the mean. Stars indicate significant differences with Ctrl 5N (t-test, n= 4-6), and levels of significance for media (Med), experiment (Exp) and their interaction (Med*Exp) effects from ANOVA (full ANOVA results are shown in **Supplementary Table 1**); ns p-value >0.05, ***p-value< 0.001.

**Figure 2:**
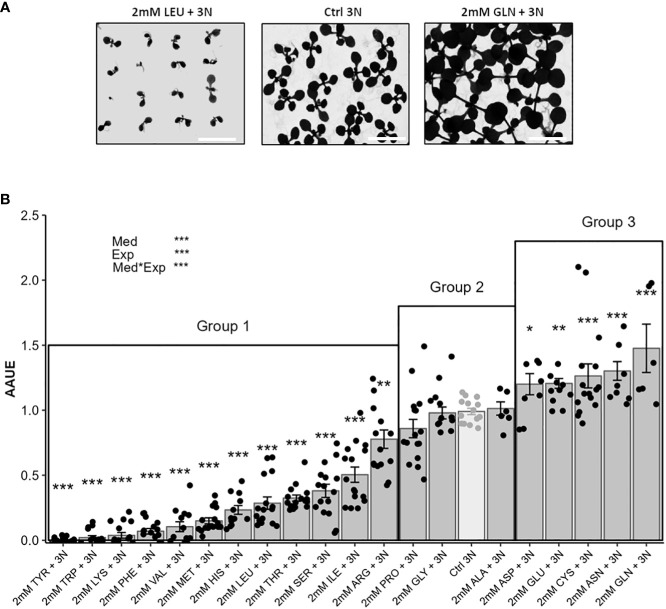
The new index “Amino acid use efficiency” (AAUE) distinguish inhibitory, neutral and beneficial amino acid nitrogen sources according to plant growth. Eighty seedlings of the Col-0 accession were grown for 12 days on an agar medium containing 1% sucrose, 3 mM KNO_3_, and 2 mM of one of the 20 proteinogenic amino acids. The controls consisted of agar medium containing 1% sucrose with 3 mM KNO_3_ (Ctrl 3N, light grey). Representative illustration of growth phenotypes for two amino acids presenting inhibitory or stimulating effects **(A)**, scale = 1.3 cm. AAUE were calculated from the leaf areas of plants grown on the different amino acid containing media according to Materiel and Methods **(B)**. Data are mean values obtained in 8 independent experiments containing 0-2 repeats. Error bars indicate the standard error of the mean. AAUE classifies amino acids into three groups: Group 1 (AAUE<1); group 2 (AAUE= 1); group 3 (AAUE> 1). Stars indicate significant differences with the Ctrl 3N (t-test, n= 6-16), and levels of significance for media (Med), experiment (Exp) and their interaction (Med*Exp) effects from ANOVA (full ANOVA results are shown in **Supplementary Table 1**); *p-value<0.05, **p-value<0.01, ***p-value<0.001.

Two-way ANOVA (R software package) was used to assess the effects of experiment (Exp), media (Med) and their interaction factors (Med×Exp) on the trait variation. Med is the media tested in the plates (different nitrogen sources), Exp is a batch of plates tested on the same date and Med×Exp is an interaction between the two main factors. Before statistical analysis, the homogeneity of variance and normality of distribution of data were tested. For **Figures 1**, **2**, these data did not fulfil the normality and homoscedasticity hypotheses, thus we used a log(x) and log(x+1) transformation respectively of data before running ANOVA with the *lm* function. Each contrast between the control condition and the other media conditions was tested by using marginal means with the function *contrast* of the R package “emmeans”. Bar plots and curves were generated in R (R version 4.2.1). Pairwise differences between conditions were carried out by t-test using Bonferroni correction to adjust multiple comparisons (p value< 0.05, with n the total number of values per condition).

## Results

### Amino acids as nitrogen source for plant growth are less efficient than nitrate

To investigate amino acids as nitrogen sources for plant growth, we measured leaf area on seedlings grown for 12 days *in vitro* on agar media containing either 5 mM of one of the twenty proteinogenic amino acids, or 5 mM of KNO_3_ as a control condition (Ctrl 5N). The use of amino acids as nitrogen source to sustain plant growth and metabolism is complex. It depends on the capacity of each amino acid to be absorbed at root level, mobilized in the plant tissue and then catabolized or use by amino-transferases. We then decided to start our study by providing each amino acid at equal molarity without considering their nitrogen stoichiometries.

We observed that the growth of the Arabidopsis seedlings was significantly reduced on amino acid containing media compared to Ctrl 5N, except in the case of glutamine (Gln), as illustrated by **Figure 1A**. In our experimental conditions, Gln was the only amino acid to promote plant growth to the same level as Ctrl 5N (**Figure 1B**). Leaf area was reduced by 52% compared with Ctrl 5N when the only nitrogen sources were glutamate (Glu) or alanine (Ala). With asparagine (Asn), aspartate (Asp), glycine (Gly), arginine (Arg), cysteine (Cys) and proline (Pro), the reduction of leaf area ranged between 69% to 88% of Ctrl 5N. Isoleucine (Ile), histidine (His), serine (Ser), and threonine (Thr) were the worst nitrogen sources as leaf area was reduced by at least 99% compared to Ctrl 5N. Seedlings barely developed after seed germination on media containing tyrosine (Tyr), valine (Val), methionine (Met), tryptophan (Trp), lysine (Lys), leucine (Leu) or phenylalanine (Phe) (**Figure 1B**). Altogether results show that except for glutamine, all the other amino acids are not a good source of nitrogen for the Arabidopsis seedling growth. The growth on amino acid media is independent of the N stoichiometry. For example, growth on Arginine, which has four nitrogen atoms per molecule, was 44% of that obtained with alanine that provides only one nitrogen atom per molecule.

### Detecting inhibitory, neutral, and beneficial effects of amino acid supplies on plant growth in the presence of nitrate using amino acid use efficiency indicator

Since we found that none of the twenty amino acids were as good nitrogen sources as nitrate for plant growth, we then questioned about the potential stimulating effects of individual amino acids on growth when nitrate is sufficient in growth medium. To test how amino acids could interfere with plant growth, we used a new agar medium that combined each amino acid (2 mM) with KNO_3_ (3 mM). Contrasted growth rates were then observed depending on the nature of the amino acid. For example, **Figure 2A** illustrates the opposite effects of leucine and glutamine on plant growth by comparison to KNO_3_ (3 mM; Ctrl 3N). Leaf areas were measured like in **Figure 1**, and we defined the “Amino acid use efficiency” (AAUE) index as the indicator of the relative plant growth on [nitrate plus amino acid] relative to [nitrate alone] [see Material and Methods, **Equations 1**–**3**)]. AAUE determined for each media allowed us to distinguish three different groups of amino acids (**Figure 2B**). Group 1 gathers the majority of the amino acids (12 in total) with AAUE significantly lower than 1. Growths on Group 1 media were decreased by 23% for Arg up to 100% for Tyr compared to Ctrl 3N (**Figure 2B**). Group 2 contains the three amino acids Pro, Gly, and Ala, and is characterized by an AAUE equivalent to 1, meaning that the presence of these amino acids in the growth medium was neutral and did not improve or reduce plant growth compared to Ctrl 3N (**Figure 2B**). The presence of amino acids from Group 3 (Asp, Glu, Cys, Asn, and Gln) is beneficial to plant growth relative to Ctrl 3N, as shown by their AAUE significantly higher than 1 (**Figure 2B**). AAUE was increased by 19% for Asp and up to 46% for Gln relative to Ctrl 3N. As Groups 1 and 2 amino acids did not stimulate plant growth under our conditions, by contrast with group 3, we decided to focus on the characterization of group 3 amino acids.

To better evaluate AAUE of the potential beneficial AA (group 3), we introduced Ctrl 5N (5 mM KNO_3_) as a new nitrate control condition and decreased the density of plants. Ctrl 5N provided the same nitrogen concentration (stoichiometry) as the [2 mM amino acid + 3 mM nitrate] condition when using Asp, Glu and Cys. Comparing the AAUE of the group 3 amino acids to the AAUE of Ctrl 5N, we found that only AAUE of Asn and Gln were significantly higher than the AAUE of Ctrl 5N (20% and 28% increase respectively; **Supplementary Figures S1A, B**). We concluded that Asp, Glu, Cys had no biostimulant effect in our experimental design.

### Bio-stimulating effects of asparagine and glutamine on plant growth are independent of enantiomeric forms and can be observed at low concentrations

**Figure 3:**
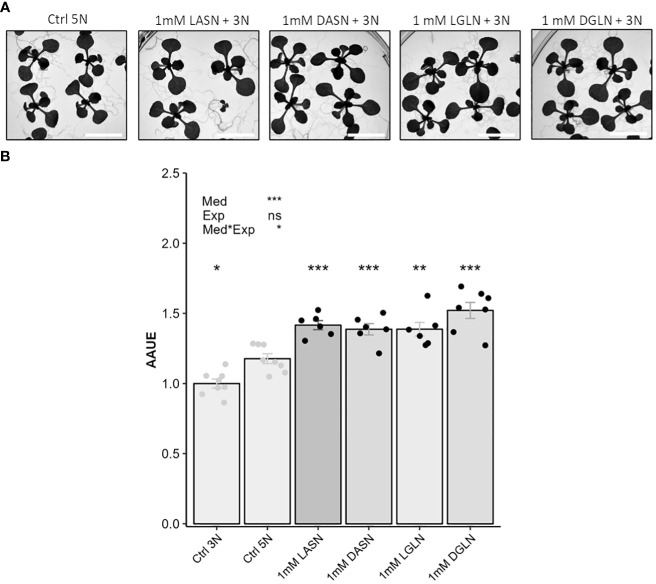
Both L and D enantiomers of asparagine and glutamine stimulate plant growth in presence of KNO_3_. Scale = 1.3 cm. In this experiment 16 seedlings of Col-0 accession were grown for 14 days on an agar medium containing 1% sucrose, 3 mM KNO_3_ and 1 mM of asparagine or glutamine. Control consists of an agar medium containing 1% sucrose and 3 mM (Ctrl 3N, light grey) or 5 mM of KNO_3_ (Ctrl 5N, light grey). Note that adding 1 mM of Asn or Gln to 3 mM nitrate medium results in 5N nitrogen stoichiometry as found in the 5 mM nitrate control. Representative pictures of plant growth **(A)**. Plant growth estimated using AAUE index **(B)**. Data represent mean values obtained in 2 independent experiments containing 3 or 4 repeats. Error bars indicate the standard error of the mean. Stars indicate significant differences with the Ctrl 5N (t-test, n= 6-8), and levels of significance for media (Med), experiment (Exp) and their interaction (Med*Exp) effects from ANOVA (full ANOVA results are shown in **Supplementary Table 1**); ns p-value > 0.05, *p-value<0.01, **p-value<0.01, ***p-value<0.001.

**Figure 4:**
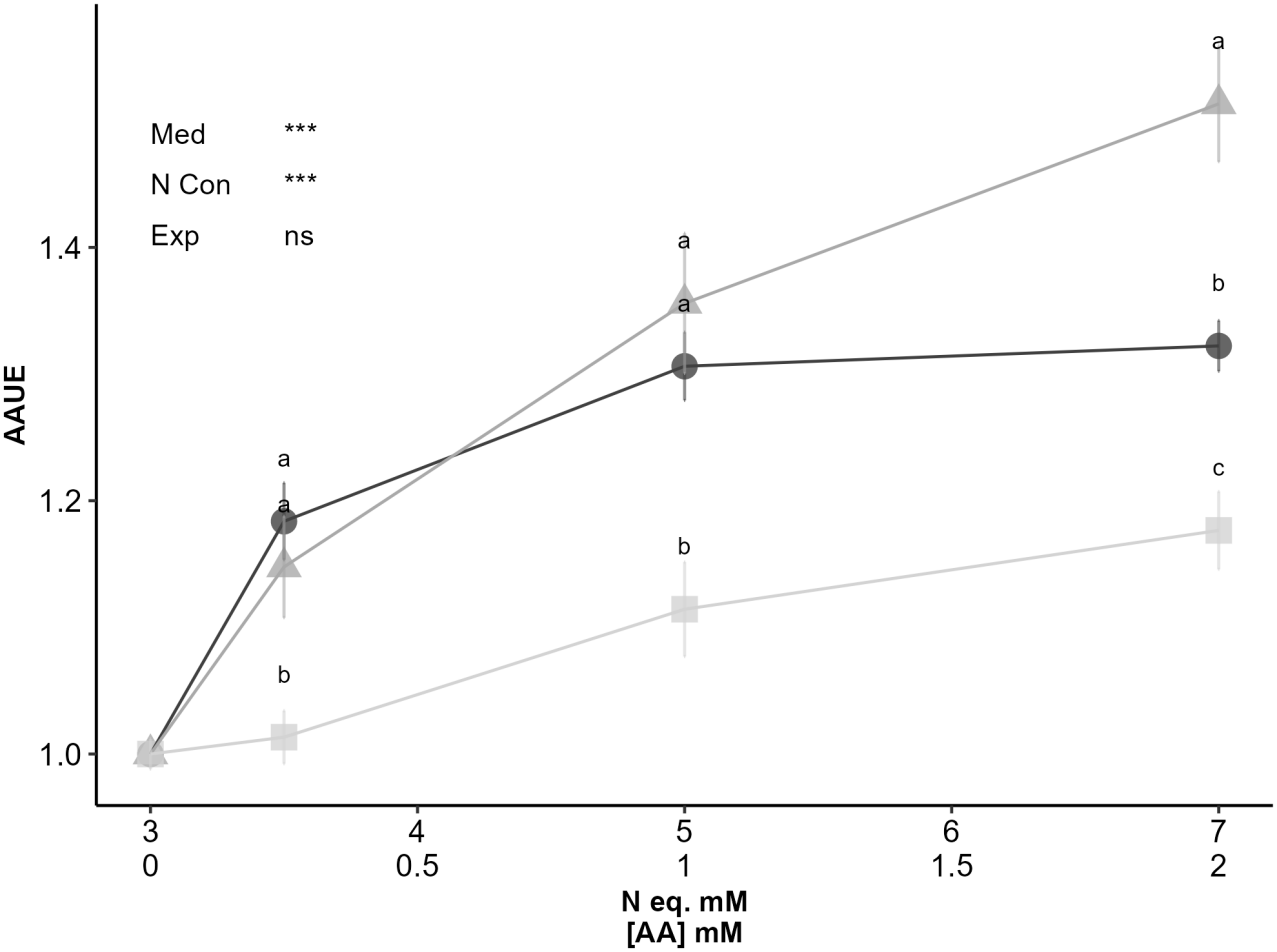
Asn and Gln stimulate plant growth at lower concentration. In this experiment, 16 seedlings of Col-0 accession were grown for 14 days on an agar medium containing 1% sucrose, 3 mM KNO_3_ and 0.00, 0.25, 1.00 or 2.00 mM asparagine (dark grey circle) or glutamine (grey triangle). N equivalent controls consisted in 1% sucrose media with 3.00, 3.50, 5.00 and 7.00 mM of KNO_3_ (light grey square). Both total N concentrations (top) and Asn and Gln concentrations (down) are presented on the X axis. Data represent the AAUE mean values obtained in 2 independent experiments containing 5-8 repeats. Error bars indicate the standard error of the mean. Different letters indicate significant differences between the AAUE obtained on the three media with equivalent N concentration (p-value<0.05, t.test, n=10-16). Stars indicate levels of significance for media (Med), N concentration (N Conc) and experiment (Exp) effects from ANOVA (full ANOVA results are shown in **Supplementary Table 1**); ns p-value>0.05, *** p-value<0.001.

To investigate the potential bio-stimulating effects of Asn and Gln on plant growth, we decided to (i) compare the enantiomeric L and D forms and (ii) to decrease the concentrations of Asn and Gln in growth media. In the experiment testing enantiomeric forms, the concentrations of the Gln and Asn were decreased to 1 mM to reach the same nitrogen stoichiometry as Ctrl 5N. The AAUE of the Asn (1 mM) and Gln (1 mM) L and D enantiomeric forms were then compared to Ctrl 5N. The AAUE of L-Asn, D-Asn, L-Gln and D-Gln were all significantly higher than the AAUE of Ctrl 5N (**Figures 3A, B**). This indicated that the bio-stimulant action of Asn and Gln was independent of the enantiomeric forms and thus independent of the possible assimilation and use in plant metabolism of these molecules. An experiment using lower concentration of L-Asn and L-Gln showed that adding 0.25 mM of one of these amino acids (1/12^th^ of the nitrate concentration) was enough to provide a positive effect on plant growth compared to the N equivalent control (**Figure 4**). This emphasizes the potential of Asn and Gln as bio-stimulants of leaf area development.

## Discussion

PHs have been described in the literature to promote plant growth and plant fitness of crops in the field (Ertani et al., 2013; Santi et al., 2017). In this study, we aimed at deciphering the role of each proteinogenic amino acid as a bioactive molecule that could stimulate plant growth and contribute to the PHs biostimulating effects. In our experimental condition, none of the twenty proteinogenic amino acids could better satisfy seedling nitrogen demand than nitrate, when used as the sole source of nitrogen. Our results were consistent with Forsum et al. in 2008, who tested ten amino acids as the sole source of nitrogen and showed that none of them were as effective as nitrate. To evaluate whether proteinogenic amino acids could have different effects on plant growth in the presence of nitrate, we then developed a new index called Amino acid use efficiency (AAUE) that was based on leaf area measurements.

AAUE facilitated the identification of amino acids providing inhibiting, neutral or stimulating effects on seedling growth when added to media containing nitrate. Among the twenty proteinogenic amino acids, we identified twelve amino acids that behaved as growth inhibitors, and three amino acids with neutral effect on plant growth, according to our experimental design. Inhibiting effects of several amino acids have already been identified, as for example in the case of branched chain amino acids, which inhibit plant growth as they have negative feedback on the synthesis of the other branched chain amino acids (Binder, 2010; Xing and Last, 2017). The feedback-inhibition of aspartate kinase by Lys, which is blocking the entrance enzyme into the Asp pathway, may also explain the negative effect of lysine on plant growth (Yang and Ludewig, 2014). Basic-, hydroxyl- and sulfur-containing amino acids were shown to severely block primary root growth at least when provided as sole source of nitrogen (Yang and Ludewig, 2014).

Regarding neutral effect, it was not surprising to find proline, that was a mediocre nitrogen source in our first experiment (**Figure 1**). More unexpected was the neutral effect of alanine that was one of the best nitrogen sources in **Figure 1**. Besides inhibitory and neutral amino acids, the five candidates (Glu, Asp, Cys, Asn, and Gln) displaying positive effects on plant growth were considered for better characterization. Taking into account nitrogen stoichiometry, determination of AAUE eliminated Glu, Asp and Cys from the potential biostimulating amino acids and led us to focus on Asn and Gln. The biostimulant properties of Gln and Asn were then completed showing that both amino acids can stimulate plant growth at low concentrations (0.25 mM; 1/12th of the nitrate supply) (**Figure 4**) thus independently of a potential carbon bonus effect. The absence of potential carbon bonus effect in our experiment was supported by the lack of growth stimulation by Asp and Glu that are built on the same carbon backbone as Gln and Asn.

In plants, amino acids are mainly present as L- enantiomeric forms. Several reports show that plants can uptake D- enantiomeric forms when available in the soil or growth medium (Bollard, 1966; Forsum, 2016). However, if and how plants utilize the D-amino acid forms remains debated and largely unclear. Gördes et al. (2011) and Gördes et al. (2013) showed that most of the D-amino acids can be absorbed by Arabidopsis seedlings. Whether racemisation of D amino acid occurs *in planta* remained unclear, but authors showed that most of the D-AAs could be metabolized and form D-Glu and D-Ala. The fact that L and D enantiomeric forms of Asn and Gln could stimulate plant growth to the same level in presence of nitrate (**Figure 3**) led us to conclude to their biostimulant properties.

Positive effects of Asn and Gln on plant growth have already been reported but without considering explicitly biostimulant effects. For instance, it was shown that supplying asparagine and glutamine at 1 mM could increase shoot length of *Phaseolus vulgaris* (Haroun et al., 2010). Glutamine application was reported to increase maize shoot dry weight by 7% (Hassan et al., 2020).

Processes for PHs production through chemical hydrolysis leads to the total conversion of asparagine and glutamine into Asp and Glu (Rouphael et al., 2020). While several of the inhibiting amino acids identified in our study are present in PHs, the fact that PHs can enhance plant growth suggests that mixtures of inhibitory, neutral and stimulating amino acid can mitigate the effects of inhibitory amino acids and possibly facilitate the expression of biostimulant effects that are independent from Asn and Gln. Accordingly, Bonner et al. (1992) demonstrated that the combination of amino acids could overcome amino acid inhibition. For instance, the inhibition of plant growth by glycine, alanine, proline and asparagine were partially antagonized by glutamine in woodland tobacco (Bonner et al., 1992). Correlative studies comparing PHs amino acid composition and biostimulant efficiency would be interesting to study and improve commercialized PHs. Such studies should also consider the supplementation of PHs with Gln and Asn regarding the biostimulant properties demonstrated here. In that context new sourcing of raw materials rich in these two amino acids has to be discovered. One other issue of PHs study is the racemisation of amino acids and the role of enantiomeric forms (Cavani et al., 2003). In the case of Gln and Asn, our study nicely shows that both the L and D enantiomeric forms display biostimulating effects.

In conclusion our study shows stimulating effect on Arabidopsis only for Gln and Asn. We could not identify biostimulating effect for any other amino acids composing protein hydrolysates. Then, our study cannot explain the biostimulating effect of protein hydrolysates on plant growth by the property of only one of the individual amino acids from their formula. Nevertheless, our *in vitro* system and AAUE index offer a new tool to estimate quantitatively the biostimulating effects of any kind of compounds. It would be now of interest to test amino acid mixtures. How our experimental design and index can be adapted to different plant species is also an interesting development. It would elucidate the biostimulant × plant species interaction, which is crucial for selecting the best combinations of amino acids depending on crops.

## Data availability statement

The raw data supporting the conclusions of this article will be made available by the authors, without undue reservation.

## Author contributions

ML: Conceptualization, Methodology, Formal analysis, Investigation, Writing – original draft. AM: Conceptualization, Methodology, Project administration, Validation, Writing – review & editing. NB-B: Writing – review & editing, Investigation. QC: Funding acquisition, Project administration, Supervision, Writing – review & editing, Conceptualization, Investigation, Methodology, Validation, Visualization. BB: Writing – review & editing, Funding acquisition, Project administration, Resources, Supervision. FC: Conceptualization, Funding acquisition, Investigation, Methodology, Project administration, Supervision, Validation, Visualization, Writing – review & editing, Formal analysis, Resources. CM-D: Conceptualization, Funding acquisition, Investigation, Methodology, Project administration, Supervision, Validation, Visualization, Writing – review & editing.
