# Supplementary material for: Discovery of the biostimulant effect of asparagine and glutamine on plant growth in Arabidopsis thaliana
Source: Front Plant Sci. 2024 Jan 22;14:1281495. doi: 10.3389/fpls.2023.1281495 (PMC10839965; doi:10.3389/fpls.2023.1281495)
Supplement: Supplementary file 1 [file Image_1.pdf]

(A)

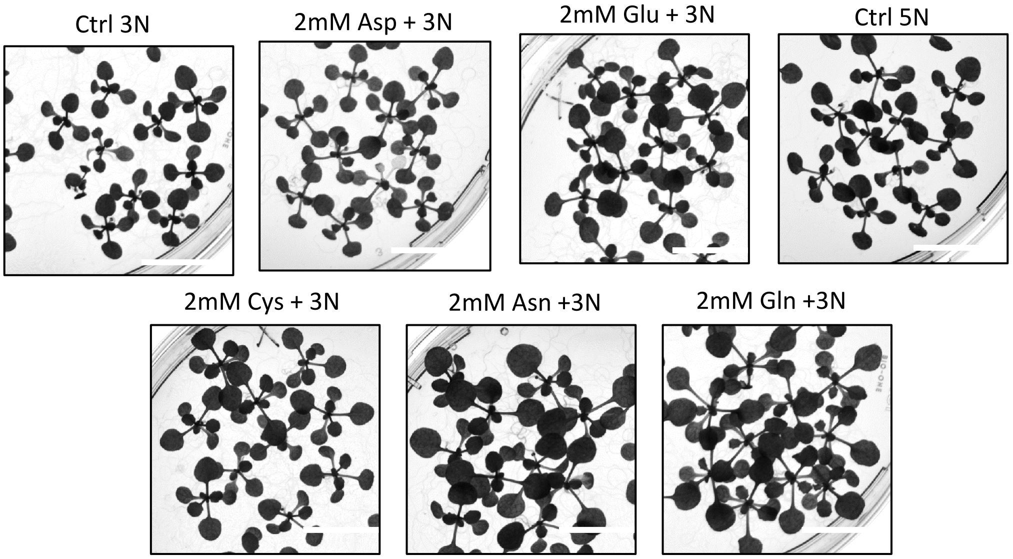

(B)

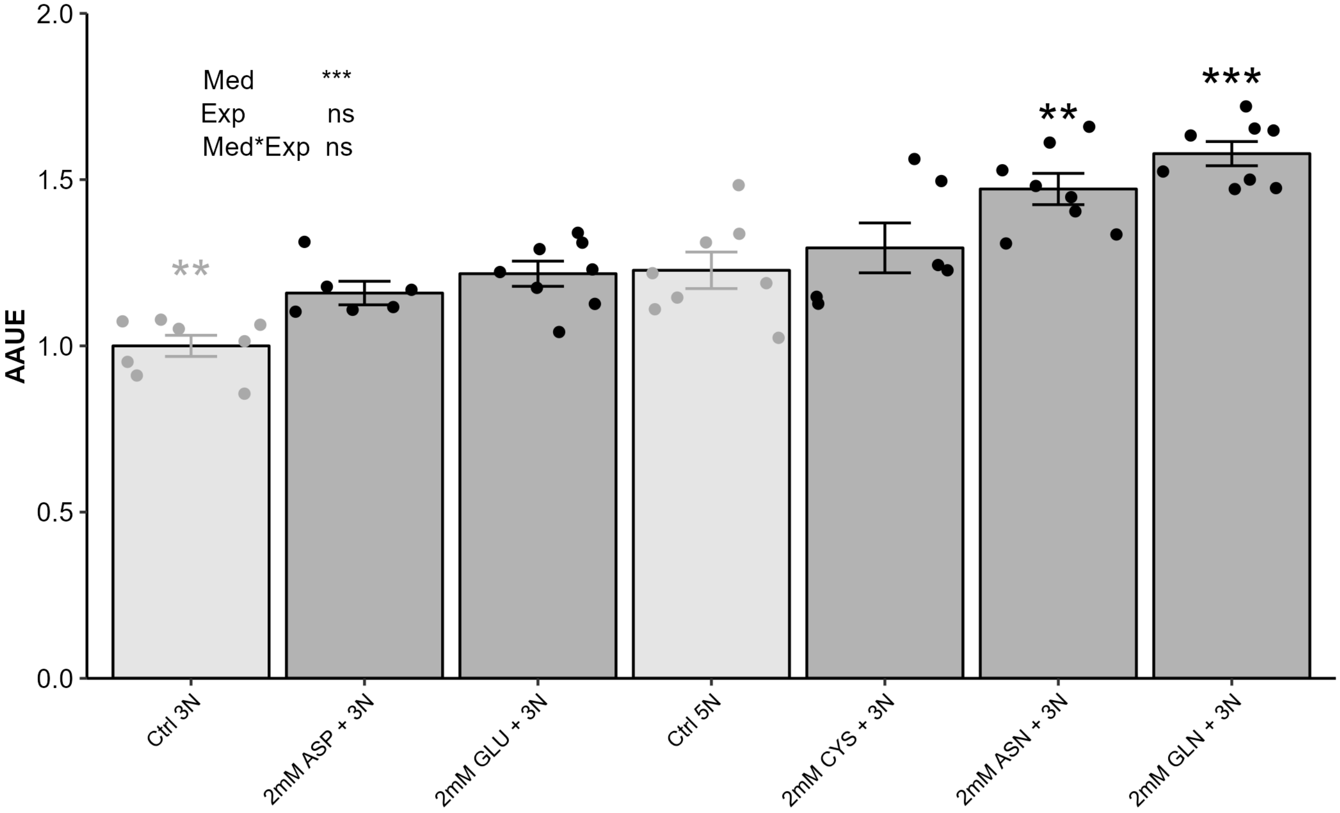

Figure S1: ASP, GLU and CYS do not stimulate plant growth compared to 5 mM nitrate. In this experiment 40 seedlings of the Col-0 accession were grown for 14 days on agar medium containing 1% sucrose, 3 mM KNO_3 and 2 mM of one of the amino acids of group 3 identified in Figure 2. Representative illustrations of growth phenotypes are presented **(A),** scale = 1.3 cm. AAUE was calculated from the leaf areas of plants grown on the different amino acid containing media according to Materiel and Methods **(B).** Two controls consists of agar media containing 1% sucrose, 3 mM KNO_3 (Ctrl 3N, light grey), or 5 mM of KNO_3 (Ctrl 5N, light grey). Data represent mean values obtained in 2 independent experiments containing 3-4 repeats. Error bars indicate the standard error of the mean. Stars indicate significant differences with Ctrl 5N (t-test, n=6-8), and levels of significance for media (Med), experiment (Exp) and their interaction (Med*Exp) effects from ANOVA (full ANOVA results are shown in supplementary Table 1); (ns p-value > 0.05 , **p-value<0.01,***p-value<0.001.
