# Supplementary material for: Discovery of the biostimulant effect of asparagine and glutamine on plant growth in Arabidopsis thaliana
Source: Front Plant Sci. 2024 Jan 22;14:1281495. doi: 10.3389/fpls.2023.1281495 (PMC10839965; doi:10.3389/fpls.2023.1281495)
Supplement: Supplementary file 2 [file Table_1.docx]

**Table S1 :** Full ANOVA results of data used in figures.
